# Size-controllable carbon spheres doped Ni (II) for enhancing the catalytic oxidation of methanol

**DOI:** 10.3906/kim-2009-77

**Published:** 2021-02-17

**Authors:** Yifen HU, Pengyu GAO, Zhen XU, Chuan ZHANG, Lizhen HUANG, Yunting HU, Yarui AN, Yingying GU

**Affiliations:** 1 Department of Chemistry, College of Science, University of Shanghai for Science and Technology, Shanghai China

**Keywords:** Methanol oxidation reaction, size-controllable carbon spheres, nickel-based materials

## Abstract

Ni(II)/CSs were prepared using a simple two-step hydrothermal method. The morphology and composition of the catalysts were studied with scanning electron microscope, transmission electron microscope, and X-ray diffraction. Fourier transform infrared spectroscopy and X-ray photoelectron spectroscopy showed that the surface of the prepared carbon spheres was rich in hydroxyl groups, which was beneficial to remove CO intermediates, and therefore, improving the catalytic efficiency and the antipoisoning ability of the catalysts. The results of cyclic voltammetry and chronoamperometry showed that the electrocatalytic activity and stability of Ni(II)/CSs were higher than that of unloaded NiAc under alkaline environment. When the nickel content was 5 wt.%, the peak oxidation current density of methanol on Ni(II)/CSs electrocatalyst reached the maximum of 34.54 mA/cm^2^, which was about 1.8 times that of unloaded NiAc. These results indicate that Ni(II)/CSs has potential applications in the electrocatalytic oxidation of methanol.

## 1. Introduction

Energy is the driving force for the development of a country’s industrialization. The large-scale use of traditional energy has led to serious environmental problems and energy crises. Therefore, it is urgent to develop new types of energy and improve energy conversion efficiency. Among various energy conversion devices, high-efficiency fuel cells have attracted widespread attention [1–3]. Methanol is a liquid fuel with high energy density, which has the advantages of abundant reserves, convenient storage and transportation, and high energy conversion efficiency. Therefore, direct methanol fuel cells (DMFCs) is one of the most promising fuel cells [4,5]. At present, the development of DMFCs faces barriers such as high catalyst costs and low methanol oxidation efficiency. In order to improve the activity of DMFCs, further research is needed to solve these problems [6–8]. The anode catalyst plays a decisive role in the performance of DMFCs [9]. In the past few decades, people have conducted extensive research on the electrocatalytic oxidation of methanol molecules on Pt-based catalysts. However, there are still some shortcomings in Pt-based materials, such as high cost and being prone to surface poisoning resulting in decreased activity, which hinders its application in DMFCs [10–12]. 

In order to reduce preparation cost of anode catalysts, many researchers have turned their attention to nonprecious metal materials, such as Mo, Ce, Mn, Co, and Ni [13–16]. These metals and their oxides and corresponding metal-based composite materials have been extensively studied; among these, nickel is a kind of metal with abundant reserves and low price [17–19]. At the same time, nickel-based materials have high electrocatalytic activity for the oxidation of small organic molecules in alkaline environments, which is due to the formation of nickel (oxy) hydroxide (NiOOH) thin layer, on which the transformation process of Ni(OH)_2_/Ni(OOH) leads to improve the electrocatalytic performance [20,21]. Therefore, nickel-based catalysts are considered to be the most valuable non-Pt-based alkaline DMFC catalytic materials.

In order to improve the activity of the anode catalyst, it is a practicable method to load the catalytic particles on the carrier. Carbon materials are the most widely used fuel cell catalyst support in current research due to their good conductivity and high stability. Carbon nanotubes [22,23], carbon nanofibers [24,25], and carbon spheres [26–28] as Pt-based electrocatalyst supports are widely used in the oxidation of methanol. In order to enhance the performance of carbon materials, researchers have paid great attention to the control of carbon nanostructures, such as surface modification and spatial structure. Therefore, it is very important to find a suitable method to prepare high-performance carbon nanomaterials. Among many synthesis methods, the hydrothermal carbonization technology using biomass resources as raw materials is a simple, inexpensive, and environmentally friendly method [12,29–32]. A large number of studies have been conducted on the synthesis of carbon spheres by hydrothermal carbonization, such as the structure control of carbon materials [33–35], the formation mechanism of carbon spheres [12,32–34], the types of biomass resources [34,35], and their application in energy storage devices [31,36,37]. Through the hydrothermal carbonization process, a large number of oxygen-containing functional groups are generated on the outer surface of carbon spheres [38]. During the methanol oxidation reaction, the introduction of these oxygen-containing functional groups may quickly eliminate the poisoning intermediate species [39].

 Accordingly, a new electrocatalyst of oxygen-containing functional groups riched carbon spheres supported Ni(II) materials as high-efficiency methanol electrooxidation catalysts were facilely synthesized by hydrothermal process. In this work, size-controlled growth of carbon spheres (CSs) was conducted by hydrothermal carbonization method. The CSs with different sizes were modified with nickel acetate to obtain Ni(II)/CSs, which were used as the catalysts for methanol oxidation. The surface of the CSs was rich in hydroxyl groups; therefore, the CSs performed both the cocatalyst and the carrier of the catalysts. The hydroxyl groups present on the surface of the carbon spheres enhance the antitoxic performance of the catalyst during the methanol oxidation process, thus improving the electrocatalytic activity and stability of the material. The Ni(II)/CSs catalysts have the advantages of simple synthesis method, low cost, and high catalytic efficiency and stability.

## 2. Experimental

### 2.1. Materials

Glucose (C_6_H_12_O_6_, Sinopharm Chemical Reagent) was used to prepare carbon spheres (CSs) templates. Nickel acetate tetrahydrate (NiAc, Ni(CH_3_COO)_2_·4H_2_O, Collins), methanol (CH_3_OH, Sinopharm Chemical Reagent), and Nafion solution (5 wt.%, Shanghai Yibang technology) were used in electrochemical testing. Anhydrous ethanol (CH_3_CH_2_OH, Sinopharm Chemical Reagent) and deionized water were used as common lotions and solvents.

### 2.2. Synthesis of CSs

Glucose was used as the carbon source. Some improvements have been made on the basis of traditional hydrothermal method to synthesize carbon spheres. In short, 6 g of glucose was dissolved in 60 mL of deionized water, and then transferred to a 100-mL Teflon-lined stainless-steel autoclave, and maintained at 180 °C for x h (x = 8,10,12,24), to get carbon spheres of different particle sizes. After the reaction was completed, the autoclave was taken out of the oven and allowed to cool to room temperature naturally. Next, the resulting black precipitate was washed with absolute alcohol and deionized water, and dried at 60 °C for 12 h.

### 2.3. Synthesis of Ni(II)/C

 Briefly, the prepared carbon spheres were dispersed in 50-mL *y* M NiAc solution ( *y* = 0.2, 0.4, 0.6, 0.8, 1.0, and 1.2). Subsequently, the suspension was transferred into an autoclave of 100 mL capacity and maintained at 70 °C for 24 h. Next, the black precipitates were collected by filtration and dried at 60 °C for 12 h to obtain the final product.

### 2.4. Material characterization

Scanning electron microscope (SEM) images and energy dispersive spectrometry (EDS) of the product were obtained with a joel 7800F microscope (Japan). Transmission electron microscope (TEM) images were taken using a FEI Tecnai G2 F20 (USA) transmission electron microscope. Bruker D8 Advance X-ray diffractometer system with Cu-K a ( l ra.15406 nm) was used to obtain X-ray diffraction (XRD) patterns. The Raman spectra were recorded using a Renishaw Raman spectrometer (UK). X-ray photoelectron spectroscopy (XPS) of the product was carried out using a Thermo ESCALAB 250XI instrument (USA). Fourier transform infrared spectrometry (FT-IR) analysis was performed by using a Thermo Nicolet 380 FT-IR spectrometer (USA).

### 2.5. u1d92 methods

A CHI660E electrochemical workstation (Chenhua, Shanghai) was used to achieve the electrochemical measurements. Three-electrode system was adopted, with platinum wire as the counter electrode, Ag/AgCl electrode as the reference electrode, and the glass carbon electrode (GCE) modified by Ni(II)/CSs nanocomposite as the working electrode. The working electrode was prepared as follows: 1 mg of catalyst and 10 μL of 5 wt.% Nafion solution were dispersed in 400 μL of absolute ethanol, and then sonicated for a few minutes to obtain catalyst ink slurry. Next, 10 uL of catalyst ink was dropped on a GCE with a diameter of 3 mm and dried in air. Thus, Ni(II)/CSs-modified GCE was obtained. Cyclic voltammetry (CV) and chronoamperometry (CA) were carried out with a scan rate of 100 mV/s at 40 °C.

## 3. Results and discussion

### 3.1. Microstructure study

#### 3.1.1. SEM

**Figure 1:**
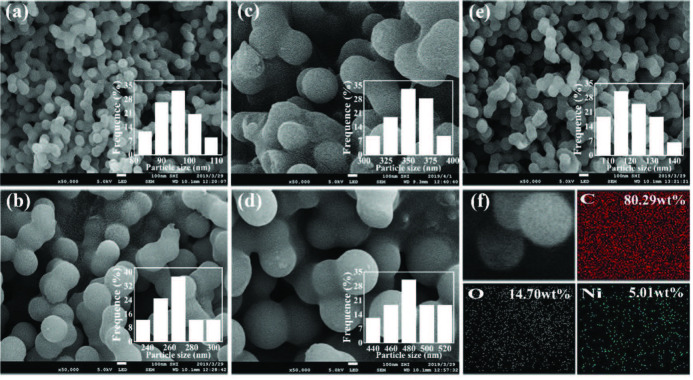
The SEM images of CSs in different sizes (a) 80–110 nm, (b) 230–300 nm, (c) 300–400 nm, and (d) 420–520 nm; (e) The SEM image of Ni(II)/CSs; (f) the area scanning element mappings of Ni(II)/CSs.

The SEM images of four carbon spheres with different particle sizes and the carbon spheres modified by Ni(II) are shown in Figure 1. In the processes of preparing carbon spheres, the hydrothermal temperature at 180 °C is higher than the glycosidation temperature, which results in aromatization and carbonization. The processes of aromatization and carbonization will be promoted as the extension of the reaction time, thus leading to the increase of the size of the carbon spheres [32]. Figure 1a displays the carbon spheres, with a fairly uniform diameter of about 80–110 nm, that are bonded to each other. With the increase of reaction time, the carbon spheres grow to about 230–300 nm in diameter, as shown in Figure 1b. Similarly, carbon spheres of 300–400 nm and 420–520 nm of u1d95 diameter can be clearly seen in Figures 1c and 1d. As the diameter of the carbon sphere grows, some of the spheres break. In Figure 1e, it can be seen that the diameter of carbon spheres is slightly larger, 90–140 nm, after the doping of Ni(II) due to the secondary hydrothermal reaction during the modification process. The EDS mappings are taken from the area in Figure 1f, in which C, O, and Ni elements with uniform dispersion are detected and the mass percentages of C, O, and Ni are 80.29%, 14.70%, and 5.01%, respectively.

#### 3.1.3. TEM

**Figure 2:**
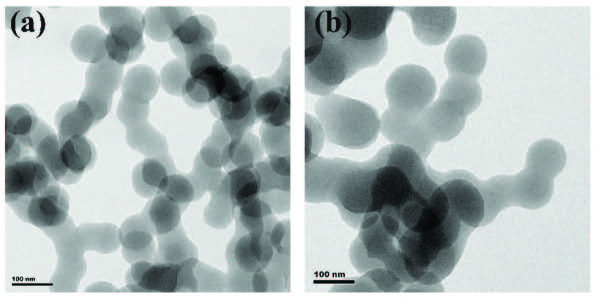
The TEM images of (a) CSs about 100 nm in diameter; (b) Ni(II)/CSs.

The TEM image (Figure 2a) shows the smallest carbon spheres, of which the diameter is about 80–110 nm, which is corresponding to the results of SEM ( Figure 1a). After loading Ni(II) on the carbon spheres, the particle size of the carbon spheres increased slightly, 90–140 nm, as shown in Figure 2b. However, the regularity decreased slightly, which matches the SEM results (Figure 1e). 

**Figure 3:**
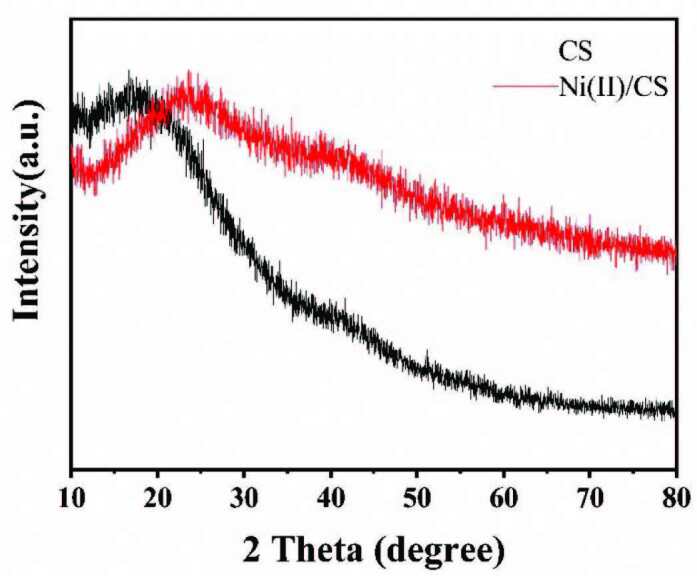
The XRD pattern of the synthesized Ni(II)/CSs.

The crystal structures of CSs and Ni(II)/CSs were measured by XRD (Figure 3). The spectra of the two samples are similar. There are two broad peaks around 20° and 44° indicating that the material corresponds to the typical amorphous carbon structure [40]. After the introduction of Ni(II), the carbon frameworks of Ni(II)/CSs are still amorphous and the diffraction peak of Ni cannot be observed in the XRD because the content of Ni in the composite material is small.

#### 3.1.4. Raman

**Figure 4:**
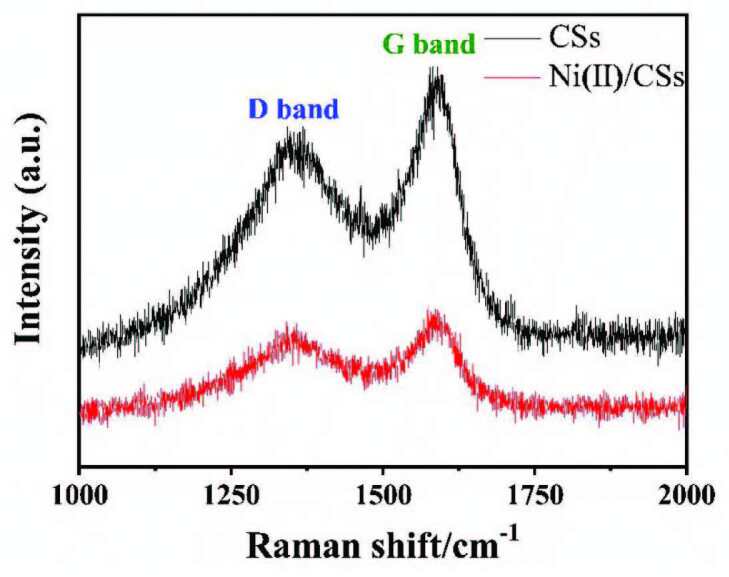
The Raman spectra of Ni(II)/CSs.

The Raman spectra of CSs and Ni(II)/CSs are shown in Figure 4. There are two obvious peaks around 1331 cm^-1^ and 1586 cm^-1^, which are assigned to the D-band and the G-band, respectively. The D-band is attributed to defects within carbon materials and the G-band represents the feature Raman-active E_2g_ mode of graphitic sheets [41]. The intensity ratio between D-band and G-band (I_D_/I_G_) of Ni(II)/CSs is 0.90, which is higher than CSs (0.83), indicating a higher degree of carbon defects in Ni(II)/CSs composites [42]. 

#### 3.1.5. FT-IR

**Figure 5:**
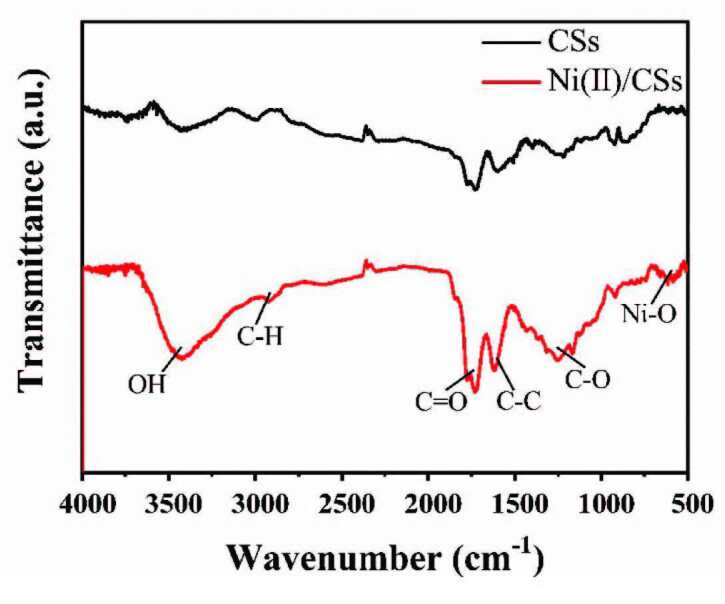
FT-IR spectra of CSs and Ni(II)/CSs.

The FT-IR spectra of pure CSs and Ni(II)/CSs are displayed in Figure 5. Similarly, in both spectrums, the broad bands above 3000 cm^-1^ due to the stretching of surface hydroxyl groups (−OH bond) [43] and the bands in the region around 3000 cm−1 arise from C−H bridging stretches. The peaks at 1760 cm−1, 1610 cm−1, and 1250 cm−1 are assigned to C=O, C-C, and C-O stretches, respectively. The presence of these functional groups on the CSs is due to the organics left from raw glucose. The spectrum of Ni(II)/CSs reveals the appearance of new band with the peak at 580 cm−1 assigned to Ni-O stretches between Ni(II) and the O on the surface of CSs [14]. 

#### 3.1.6. XPS

**Figure 6:**
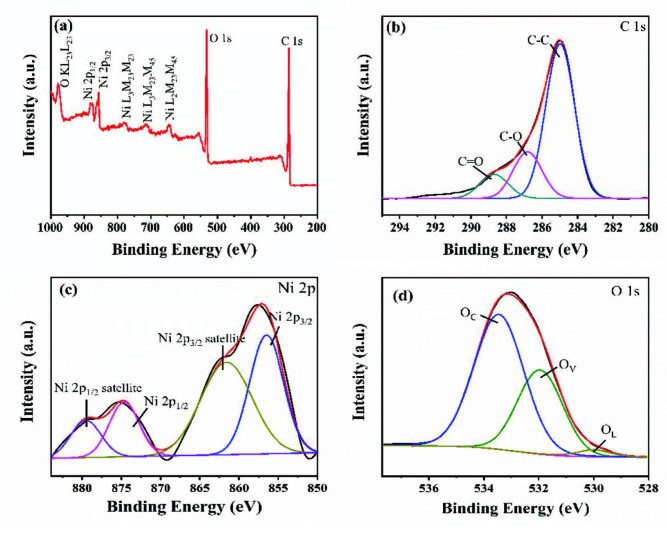
XPS spectrum of Ni(II)/CSs: (a) full-wide scanned spectrum, (b) C 1s spectra, (c) Ni 2p spectra, (d) O 1s spectra.

The surface features and atomic valence states of Ni(II)/CSs nanocomposites were characterized by XPS, as illustrated in Figures 6a–6d. The wide-range spectrum of the Ni(II)/CSs is exhibited in Figure 6a; Ni, O, and C elements can be found in Ni(II)/ CSs. As show in Figure 6b, the high-resolution C 1s spectrum of the Ni(II)/CSs can be deconvolved into three peaks for C-C, C-O, and C=O bonds that located at 284.8, 286.4, and 288.1 eV, respectively [44]. The existence of C-O and C=O bonds are attributed to the carbonyl groups and hydroxyl groups on the surface of the CSs, which matches the FT-IR results. Figure 6c displays the deconvolved spectrum of Ni 2p. The peaks with the binding energy of 856.1 and 873.8 eV belong to the Ni 2p_3/2_ and Ni 2p_1/2_ peaks of Ni(OH)_2_, respectively. The peaks located at 861.6 and 879.8 eV are satellite peaks attributed to the Ni 2p_3/2_ and Ni 2p_1/2_ peaks of NiO, respectively [45,46]. Figure 6d shows the O 1s spectra of Ni(II)/CSs. The O 1s spectrum is fitted into three peaks with the binding energies at 529.8 eV, 531.9 eV, and 532.9 eV. The peak at 529.8 eV can be assigned to the lattice oxygen (O_L_), the residual peaks at 531.9 and 532.9 eV are generally associated with oxygen vacancies (O_V_) and surface species (e.g., hydroxyl) [46].

### 3.2. Electrochemical test

#### 3.2.1. Performances of different electrodes

**Figure 7:**
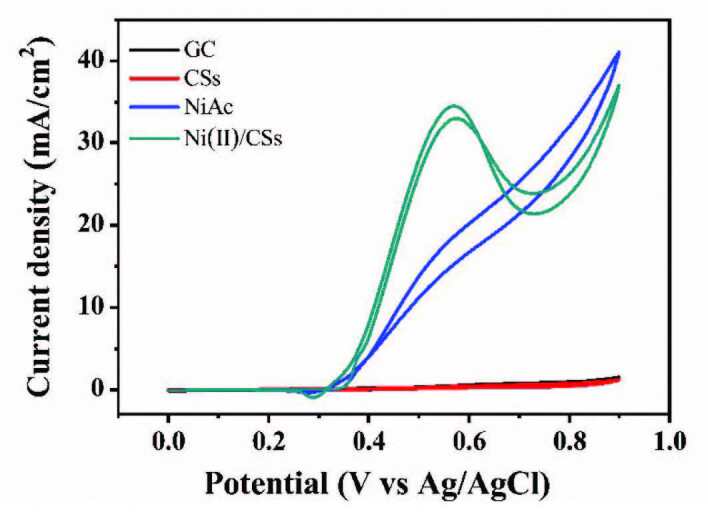
CVs of different electrodes in 1.5 M NaOH and 1.5 M CH3OH.

In order to prove the catalytic oxidation of methanol by catalysts, different electrodes were prepared for cyclic voltammogram testing, as shown in Figure 7. The bare glassy carbon electrode and the pure carbon spheres electrode have no catalytic effect. Nickel acetate electrode was also prepared, but its peak current density of methanol oxidation at the potential at 0.57 V was only about 18.64 mA/cm^2^. Compared with other electrodes, the Ni(II)/CSs electrode showed much higher activity for methanol oxidation, over which the peak current density reached about 34.54 mA/cm^2^, Obviously, the nickel-based catalysts is greatly enhanced with the support of carbon spheres. The catalytic mechanism of the anode catalyst on the oxidation reaction of methanol in an alkaline environment is as follows [47]: 

M + H_2_O → M(OH)_ads_ + H^+^ + e^-^ (1) 

NiOOH + CH_3_OH NiOOH(CO)_ads_ + H^+^ + e ^-^ (2) 

NiOOH(CO)_ads_ + NiOOH + OHads→ CO_2_ + Ni(OH)_2_ + H_2_O (3) 

The methanol molecules are adsorbed on the surface of catalyst, and then are dehydrogenated to produce intermediate species (CO)_ads_. The adsorbed CO species react with the surface hydroxyl group to form CO_2_ and release the surface catalytic active sites. The above reaction (3) is the rate determining step of methanol oxidation reaction [20]. That is to say, the content of the surface hydroxyl is one of the key points of accelerating methanol oxidation reaction. 

The mechanism of methanol oxidation over the Ni(II)/CSs catalysts has been shown in Scheme. In this process, Ni(II) is the main catalyst, catalyzing methanol dehydrogenation to generate adsorbed CO as the reaction (2) mentioned above. The carbon spheres is the cocatalyst, the hydroxyl group on the surface of the carbon spheres reacts with the adsorbed CO to convert (CO)_ads_ into CO_2_ and release the surface catalytic active sites at the same time, as the reaction (3) above. In fact, FT-IR spectra and XPS results prove the existence of surface hydroxyl groups on Ni(II)/CSs, which can remove CO intermediates and prevent catalyst poisoning, thus improving the catalytic oxidation activity of methanol.

**Scheme:**
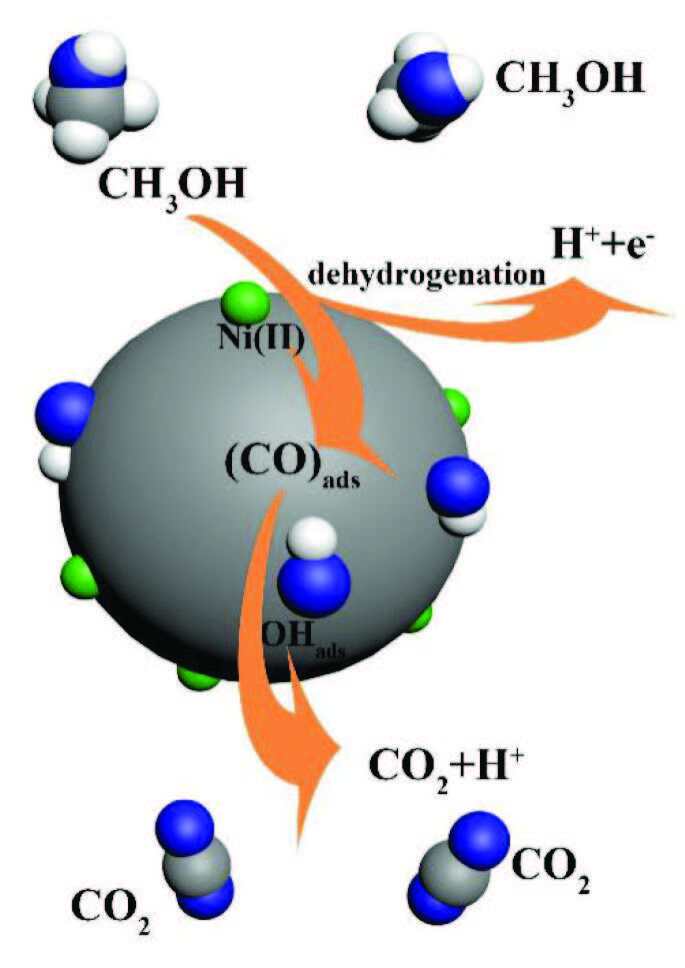
Schematic illustration for the Ni(II)/CSs enhancing the activity in MOR.

#### 3.2.2. Performance of CSs in different sizes 

**Figure 8:**
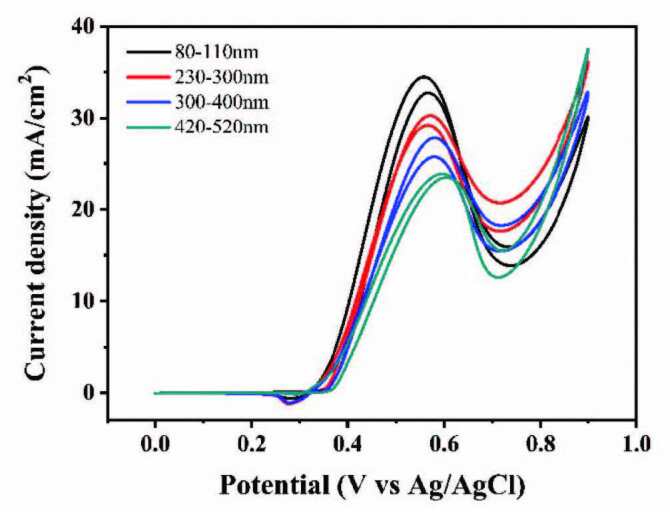
CVs of catalysts prepared from carbon spheres of different diameters in 1.5 M NaOH and 1.5 M CH3OH.

In order to explore the supporting properties of four carbon spheres of different sizes, their electrochemical properties were tested as shown in Figure 8. It can be seen that the activities of the four catalysts decrease gradually with the increase of carbon sphere diameter. This is probably the smaller the diameter of the carbon sphere, the larger the specific surface area, the stronger the catalytic effect. Therefore, the catalyst with the smallest diameter was selected for the subsequent electrochemical test.

#### 3.2.3. Performance of different scanning laps

**Figure 9:**
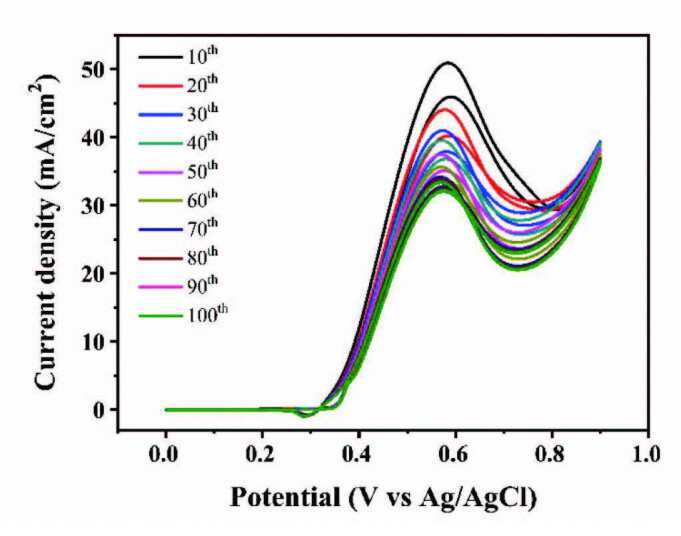
CVs of Ni(II)/CSs with different scanning laps in 1.5 M NaOH and 1.5 M CH3OH.

Under different scanning laps, Figure 9 depicts the changes of the oxidation peak current density of methanol. On 10th lap, peak current density can reach more than 42.46 mA/cm^2^. When the number of scanning laps increased from 10 to 60, the peak current density of methanol anodic oxidation gradually decreased, indicating that the methanol oxidation reaction had not reached a stable state. When the number of scanning laps changed from 60 to 100, the anodic oxidation peak current density only decreased by about 4%, indicating that the methanol oxidation reaction over the catalyst reached a relatively stable state in this stage. Therefore, the number of scanning turns is chosen to be 60 in the following electrochemical test.

#### 3.2.4. Performances of different nickel contents

**Figure 10:**
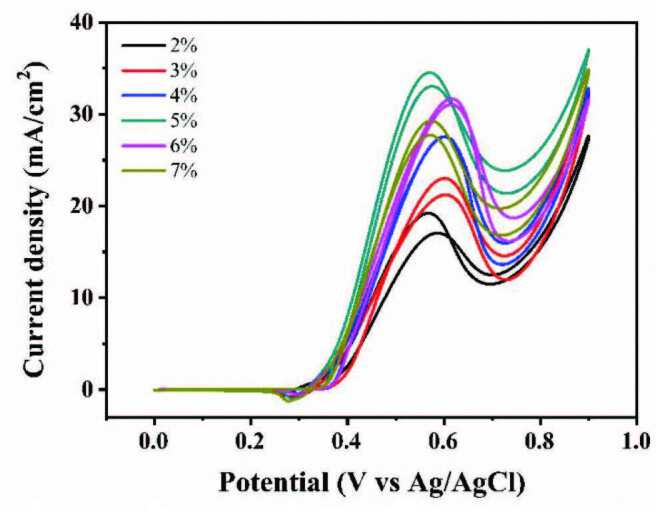
CVs of Ni(II)/CSs with different Ni contents in 1.5 M NaOH and 1.5 M CH3OH.

As shown in Figure 10, catalysts with different nickel contents also have different effects on methanol oxidation. When the nickel contents are 2% and 3%, the peak current densities are only about 19.22 mA/cm^2^ and 20.35 mA/cm^2^, respectively. The peak current density reaches a maximum of 34.54 mA/cm^2^ when nickel content is 5%. However, when the nickel content continued to increase to 6% and above, the anodic oxidation peak current density gradually decreased, which may result from CO poisoning caused by excessive Ni(II) leading to the faster dehydrogenation of methanol to form more CO intermediates without being eliminated timely by the surface hydroxyl groups.

#### 3.2.5. Performances of different methanol concentrations

**Figure 11:**
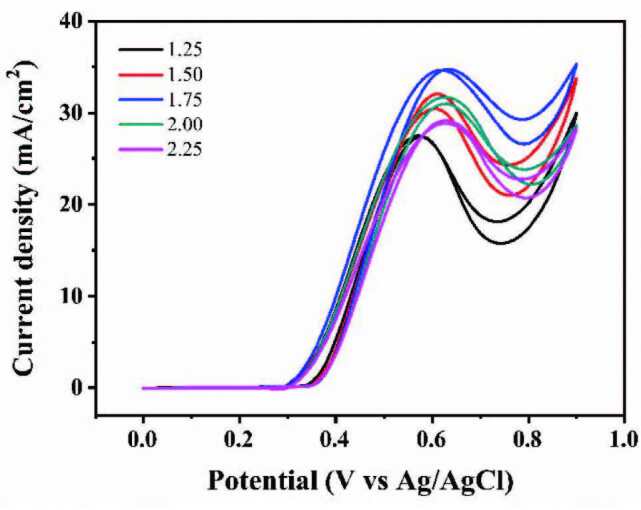
CVs of Ni(II)/CSs in solutions of 1.5 M NaOH and different CH3OH concentrations.

The composition of electrolyte is also an important factor affecting the catalytic effect of the catalyst. The content of sodium hydroxide remains unchanged and the catalytic performances with different methanol concentrations are shown in Figure 11. When the methanol concentration changes from 1.25 M to 1.75 M, the peak current density gradually increases. However, when the methanol concentration was further increased to 2.25 M, the peak current density decreased with the increase of methanol concentration. Naturally, the current density reached a maximum of 34.54 mA/cm^2^ when the concentration of methanol electrolyte was 1.75 M. It is worth noting that when the methanol electrolyte is in a high concentration (1.75–2.25 M), the peak current density decreases because excessive methanol produces too much CO intermediates and thus accelerates the catalyst poisoning.

#### 3.2.6. Performances of different sodium hydroxide concentrations

**Figure 12:**
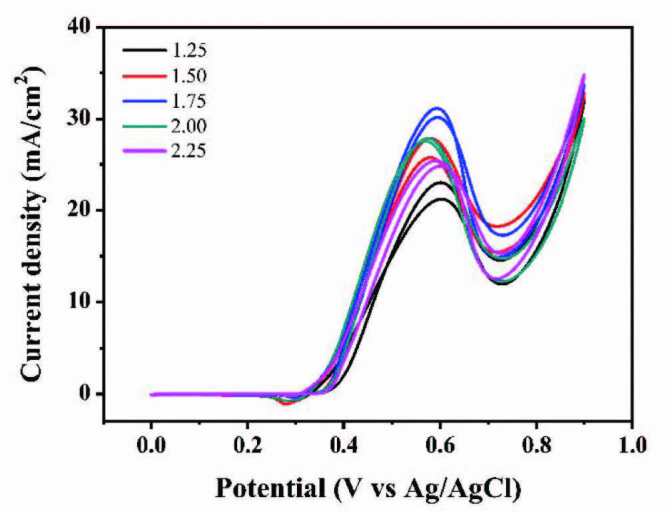
CVs of Ni(II)/CSs in solutions of 1.5 M CH3OH and different NaOH concentrations.

NaOH plays an important role in providing an alkaline environment for the catalytic oxidation of methanol. As shown in Figure 12, at the constant concentration of methanol, the peak current density gradually increases as the concentration of NaOH increases. The maximum value of the current density is 34.54 mA/cm^2^ at 1.75 M NaOH concentration. However, the current density decreases when the concentration of NaOH exceeds 1.75 M due to the competitive adsorption of OH^-^ with methanol.

#### 3.2.7. Stability

**Figure 13:**
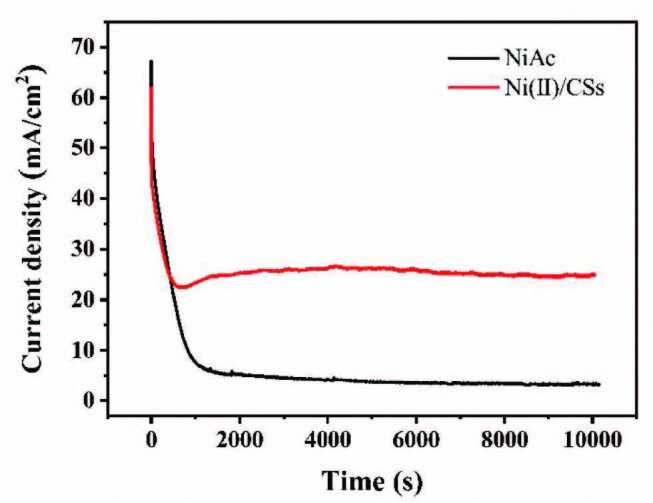
Chronoamperometric curve of NiAc and Ni(II)/CSs in 1.75 M CH3OH and 1.75 M NaOH at 0.60 V vs. Ag/AgCl.

Figure 13 shows the chronoamperometry curves for pure NiAc and Ni(II)/CSs to evaluate their stability. The test was measured at 0.6 V for 10,000 s in 1.75 M CH3OH and 1.75 M NaOH solution. It can be clearly seen from the figure that the current response of Ni(II)/CSs electrode is significantly higher than that of (CH_3_COO)_2_Ni electrode, which means the Ni(II)/CSs catalyst has higher electrocatalytic activity in methanol oxidation reaction. The high current response at the initial stage is due to the double charging process. The rapid current decrease in the following few minutes is caused by the rapid formation of poisoning intermediates of the methanol oxidation. Then the (CO)_ads_ is eliminated by the surface hydroxyl to generate CO_2_ and the reaction reaches a relatively stable state. As shown in the figure, after a long period of testing, the current over the Ni(II)/CSs catalyst keeps high and stable, which proves the highly efficient cocatalysis of the surface hydroxyl on the CSs [48]. Therefore, the results indicated that the Ni(II)/CSs have good catalytic activity and stability for methanol oxidation.

In addition, the comparison of the methanol oxidation activity between Ni(II)/CSs with the latest reported nickel-based electrocatalysts is listed in Table [49–54]. Obviously, the methanol oxidation over the NiPt-based alloy catalysts is carried out under acidic conditions. Although the catalytic activity of these materials is comparatively low, its advantage lies in the lower onset potential. The reaction over non-Pt Ni-based materials is carried out under alkaline solutions. The activities of these materials are much higher than that of NiPt-based catalysts, but their onset potentials are comparatively high. In comparison with the non-Pt catalysts, the Ni(II)/CSs catalyst has the advantages of high current density, low onset potential, and simple and low-cost synthesis method.

**Table:** Comparison of Ni(II)/CSs catalyst with other catalysts

Catalyst	Electrolyte	Current density(mA/cm^2^)	Onset potential(V)	References
Ni(II)/CSs	1.75 M NaOH	34.54 mA/cm^2^	0.3 vs. Ag/AgCl	This work
Pt3Ni	0.1 M HClO4	1.21 mA/cm^2^	0.6 vs. RHE	49
Pt94Ni6UNWs	0.5 M H2SO4	0.968 mA/cm^2^	0.25 vs. Ag/AgCl	50
Pt@mPtNiCBs	0.5 M H2SO4	1.58 mA/cm^2^	0.2 vs. Ag/AgCl	51
Ni-BTC	2 M NaOH	27.16 mA/cm^2^	0.45 vs. Hg/HgO	52
NiO NTs-400	1 M KOH	24.3 mA/cm^2^	1.33 vs. RHE	53
NiCo2O4/Ni(OH)_2_	1 M KOH	132 mA/cm^2^	0.3 vs. Ag/AgCl	54

## 4. Conclusion

Reaction time modulation of the hydrothermal carbonization process led to size-controlled growth of CSs ranging from approximately 80 to 500 nm in diameter. Ni(II)/CSs were loaded by hydrothermal treatments and used as the anode catalyst of direct methanol fuel cell. The results of SEM and TEM show the successful preparation of carbon spheres with different particle diameters and the tendency of the increase in particle size and the decrease in overall regularity of the carbon spheres after Ni(II) modification. FT-IR spectroscopy and XPS spectroscopy indicate that there were a large number of hydroxyl groups on the surface of the catalyst, which can accelerate the elimination of CO intermediates and improve the catalytic efficiency. XPS research also shows that nickel exists in the catalyst in the form of divalent nickel ions. The results of cyclic voltammetry showed that compared with the unloaded NiAc, the electrocatalytic efficiency of Ni(II)/CSs on methanol oxidation was significantly improved. This was mainly due to the synergistic effect of the hydroxyl group on the surface of the carbon sphere and Ni(II), which improved the efficient conversion of CO to CO2. The electrochemical performance test also showed that the maximum oxidation peak current density reached 34.54 mA/cm^2^ over the Ni(II)/CSs catalysts when the nickel content was 5wt% under the electrolyte solution of 1.75 M CH_3_OH and 1.75 M NaOH, and the chronoamperometry test showed that the Ni(II)/CSs had good stability. Therefore, the Ni(II)/CSs catalysts can be used as a potential catalysts for the catalytic oxidation of methanol.
